# Perceptions of community members on contextual factors driving cardiovascular disease behavioural risk in Ghana: a qualitative study

**DOI:** 10.1186/s12889-022-13646-3

**Published:** 2022-06-22

**Authors:** Naa Adjeley Mensah, Olutobi Adekunle Sanuade, Leonard Baatiema

**Affiliations:** 1 Regional Institute for Population Studies, University of Ghana, Accra, Ghana; 2 Department of Medical Social Sciences, Northwestern University Feinberg School of Medicine, Chicago, USA; 3 Department of Health Policy, Planning and Management, School of Public Health, University of Ghana, Legon, Accra, Ghana

**Keywords:** Cardiovascular disease, Behavioural risk factors, Barriers, Contextual factors, Ghana

## Abstract

**Background:**

There is clear evidence that lifestyle interventions are effective towards reducing cardiovascular risk. However, implementing these interventions in real-world setting has been suboptimal, especially in sub-Saharan Africa, thus creating ‘evidence to practice gap.’ We explore perceptions of community members on contextual factors driving cardiovascular disease (CVD) behavioural risk (alcohol consumption, smoking, physical (in)activity and fruits and vegetables consumption) in Ghana.

**Methods:**

This was a cross-sectional study. Thirty (30) focus group discussions (FGDs) were carried out in five communities in Ghana (Ga Mashie, Tafo, Gyegyeano, Chanshegu and Agorve) between October and November 2017, and these were analysed using a thematic approach.

**Results:**

Five main factors were raised by participants as contextual factors driving alcohol consumption and smoking and these include economic (poverty, unemployment, loss of jobs), psycho-social (worries, hardships, anxieties), medical (pain suppression, illness management), sexual (sexual performance boost), and socio-cultural (curse invocation, quest for supernatural powers) factors. Personal/social factors (time constraints, personal dislike, lack of knowledge of the benefits of exercise), economic factors (poverty, economic hardship), and negative health effects (getting tired easily, medical conditions) were cited as reasons why community members did not engage in physical activity. Consumption of fruits and vegetables in the five communities were determined by availability, cost, personal (dis)like, lack of knowledge about benefits, and cultural taboo. Participants’ narratives revealed that the symbolic functions of some of these behavioural risk factors and the built environment were important determining factors that have sustained these behaviours in these communities over the years.

**Conclusions:**

This study showed that successful implementation of CVD interventions in Ghana needs to address the perceptions of community members on factors driving CVD behavioural risk factors. Future policies and interventions should be developed based on these contextual factors taking into consideration the age, sex and ethnic variations especially with interventions seeking to address CVD risk factors at the primary health care level. These findings should urge local policy makers and health managers to incorporate the roles of these contextual factors in new programs targeting cardiovascular health. Closing the ‘evidence to practice’ gap as far as CVD interventions are concerned may be impossible without this.

**Supplementary Information:**

The online version contains supplementary material available at 10.1186/s12889-022-13646-3.

## Background

Globally, cardiovascular diseases (CVDs) are the number one cause of deaths. About 17.9 million people died from CVDs in 2019 and this represents 32% of global deaths [1]. CVDs are largely preventable, or can be treated, yet, both high-income countries (HICs) and low-and-middle income countries (LMICs) are yet to successfully control the rising burden of CVDs. It is also clear from previous studies that most CVDs can be prevented if major behavioural risk factors such as harmful use of alcohol, tobacco use, unhealthy diet and obesity, and physical inactivity, are addressed [1] Majority of the CVDs burden occurs in LMICs where inadequate capacity and resources to support prevention, early detection, treatment and control of CVDs is still a major challenge [2]. Although the growing burden of CVDs is a major public health challenge, resource-poor settings in sub-Saharan Africa (SSA) bear the brunt of this global CVD burden [3]. There are concerns that the growing CVDs in these settings are likely to escalate into epidemic proportions if stringent measures are not swiftly and aggressively put in place to address the burden of these diseases [4, 5].

The rising CVDs burden in SSA is underpinned by a multiplicity of risk factors. For example, risk factors such as physical inactivity, unhealthy diets, smoking, alcohol consumption and high level of cholesterol are reported to be driving the CVD epidemic in SSA [6]. As highlighted by the INTER-HEART Africa study, smoking, physical inactivity, poor dietary patterns, hypertension, high cholesterol levels accounts for about 90% of myocardial infarctions, including poor stroke outcome [7]. Although limited information exists relating to CVDs risk factors in African countries and other LMICs, the few evidence suggest physical activity levels in African countries and those from other LMICS are much lower compared to HICs [8].

Furthermore, despite technological advancement in medical practice for CVD prevention and treatment, control of CVDs in SSA is still far from optimum. Uptake of evidence-based CVD prevention and treatment interventions remain limited, and quality of CVD care is suboptimal in the region. One of the main factors driving the increasing burden of CVDs in Africa is low awareness. For instance, there is low awareness of early sign and symptoms of stroke [9] and hypertension [10] and people often report such conditions in their advanced stages [11]. A probable reason for the low CVDs awareness is the lack of public awareness campaigns on CVDs. Compared to HIC where such public awareness campaigns on CVDs exist including the UK [12] and other HICs [13], same cannot be said for countries in SSA and other LMICs. Against the background of an increasing burden of CVDs, there is the pressing and urgent need to design CVDs preventive programs and interventions in LMICs such as Ghana.

To date, some of the interventions that have been developed to minimise the behavioural risk factors include education and awareness creation programs, screening programs for CVDs risk factors including individual lifestyle change program on diet, smoking, physical activity, self-management and medication adherence, and blood pressure and weight reduction program [14–17]. Despite these interventions, few advances have been made in minimising the prevalence and incidence of CVDs most especially in SSA partly because of limited consideration for the contextual factors driving the CVDs behavioural risk factors. This study was conducted to address these gaps and explore perceptions of community members on the contextual factors (facilitators and barriers) driving selected CVD behavioural risk factors (alcohol consumption, smoking, physical (in)activity and fruits and vegetables consumption) in Ghana. This study offers insights into existing perceptions on CVDs behavioural risk factors in Ghana and the factors driving these perceptions. The evidence generated in this study could inform strategies to improve the implementation of subsequent CVD interventions in the country.

## Methods

### Study design

This was a cross-sectional study. Data were gathered using focus group discussions (FGDs) to explore the knowledge and understandings of local community residents on cardiovascular disease behavioural risk factors. Sample of the questions that were asked are provided in Table 1. The interview guide was developed based on previous qualitative studies on non-communicable diseases [18, 19]. The interview guide was piloted in similar population to check the validity and relevance of the questions. Some of the questions were re-phrased for clarity after the guide was piloted. The aim of the broader study was to explore local community perceptions on chronic illness and stroke with particular focus on: general life history; knowledge on health and illness; knowledge on definition, causes, management and prevention of chronic illness and stroke, and; perceptions on CVD behavioural risk factors (alcohol consumption, smoking, physical inactivity, and fruits and vegetables consumption) [20, 21]. We focused on these risk factors because they are the most important behavioural risk factors of CVD highlighted by the World Health Organization [1, 22].

**Table 1:** Interview guide

**Alcohol**	• What do you think causes people to consume alcohol in this community? • What types of alcohol are mostly consumed in this community*? (Explore if types of drink consumed are based on social class)* • In what ways do you think alcohol can affect the health of an individual? ◦ Do you think there is an association between alcohol consumption and chronic disease*? (Explore nature of association)* • In what circumstances do people mostly consume alcohol in this community? ◦ *Prompts: during funerals, social gatherings, job loss and other economic disruptions, marital disruption, political campaigns, *etc • What roles does alcohol perform in this community? *(Explore views on religious functions, economic functions, political functions, and social functions)*
**Smoking**	• What do you think causes people to smoke in this community? *(Explore different types of smoking)* • In what ways can smoking affect the health of an individual? • Do you think there is an association between smoking and chronic disease? *(Explore nature of association)* • In what circumstances do people mostly smoke in this community? ◦ *Probes: during funerals, social gatherings, job loss and other economic disruptions, marital disruption, *etc • What roles does smoking perform in this community? *(Explore views on religious functions, economic functions, political functions, and social functions)*
**Physical activity**	• What does physical activity means to you? *(Explore if they have heard of the benefits of physical activity and find out the sources of knowledge)* • In what ways do you think an individual can be physically active? ◦ *Prompts*: through exercise, nature of jobs, etc • How will you describe the level of physical activity in this community? • In your opinion, is there an association between physical activity and chronic diseases? *(Explore nature of association)* • What do you think hinders people in this community not to be physically active? ◦ *Prompts*: lack of physical activity space, nature of jobs, non-relevance to culture, etc
**Fruits and vegetables**	• What types of fruits are available in this community? *(Explore list of fruits)* ◦ What fruits are available seasonally? ◦ What fruits are available throughout the year? ◦ Do you think fruits are easily accessible/affordable by everyone in this community? *(Explore reasons)* • What types of vegetables are being consumed in this community? *(Explore list of vegetables)* ◦ Do you think they are easily accessible/affordable by everyone in this community? *(Explore reasons)* • In what ways do you think fruits and vegetables affect the health of an individual? ◦ Is consumption of fruits and vegetables associated with chronic diseases? *(Explore reasons)* ◦ What are the other functions of fruits and vegetables? ◦ What types of health messages have you heard about benefits of fruits and vegetables? *(Explore sources of knowledge)* ◦ What hinders people in this community from adhering to such health information?

### Study site and sampling

This study was conducted in five communities across Ghana. These include Ga Mashie (Greater Accra region), Tafo (Ashanti region), Gyegyeano (Central region), Chanshegu (Northern region), and Agorve (Volta region). Ga Mashie, Gyegyeano and Agorve were in the southern part of the country; Tafo was in the middle-belt and Chanshegu in the northern part. The five communities were selected to capture variations in perceptions from major ethnic groups located in different geographical locations in Ghana. These ethnic groups include Akan (Tafo & Gyegyeano), Ga (Ga Mashie), Dagomba (Chanshegu) and Ewe (Agorve). These communities were characterised by poor economic circumstances, poor household living arrangements, low education, and low access to healthcare [18, 19]. Many of the residents in these communities were farmers and petty traders and characterised by low-income earnings.

Data were collected through FGDs. Participants were purposively recruited from these five communities, and they were aged 18 to 75 years. All the FGDs were conducted by thirteen trained research assistants, with an average of two research assistants in each of the five communities. OAS was present in all the group discussions and coordinated all research activities. In Tafo, Gyegyeano, Chansegu and Agorve, OAS and the respective research assistants approached traditional leaders and other key community leaders before recruitment of the study participants was carried out. In Ga Mashie, however, OAS had previous engagements with the community and recruitment was relatively easy.

### Data collection

The fieldwork was carried out between October and November 2017. Six (6) FGDs were conducted in each of these study sites, giving a total of 30 FGDs in the five communities and they were segmented by age and sex. The FGDs were moderated by trained research assistants who are experienced in qualitative research methodology. The FGDs were conducted in Ga (Ga Mashie), Twi (Tafo), Fante (Gyegyeano), Dagbani (Chanshegu) and Ewe (Agorve). The duration of the group discussions ranged from 90 to 150 min and permission was sought from the participants to record all the group discussions. The group discussions were composed of between six and ten participants. A total of 255 people participated in the study. The mean age of the participants was 43.4 years. More than half were females (51.7%, *n* = 132), 84.3% (*n* = 215) had secondary education or lower, and about three-quarter were involved in informal occupation such as trading and agriculture (74.9%, *n* = 191). Close to half were married (48.6%, *n* = 124) and a higher proportion were Christians (65.1%, *n* = 166). Further details about the study have been provided in other studies [20, 21]. All the participants were remunerated for participating in the study and remunerations were provided after the data collection was completed to ensure that recruitment was based on voluntary participation. Ethical approval was obtained from the University College London Research Ethics Committee (REC) with approval number 11371/001, and locally from Ghana Health Service Ethics Review Committee (GHS-ERC Number- 007/09/17).

### Data analysis

The data was analysed through a thematic approach after all the FGDs were transcribed. All the group discussions were transcribed from Ga, Twi, Fante, Ewe and Dagbani into English language by a team of transcribers with competencies in the various local languages. Coding was done using ATLAS TI 7 and data was analysed through thematic analysis. NAM and OAS were involved in the generation of codes and themes for the data. There was continuous discussion between the two authors at each stage of the coding process. The first stage of the analysis involved generating codes after familiarisation with the transcripts. This process was guided by a coding frame with two sections: 1) a section on previous existing codes derived from qualitative studies on perceptions on CVD behavioural risk factors [23] and an open-ended section of inductive codes derived from perceptions of the study participants. The second stage of the analytical framework involved identifying the linkages between codes, themes and appropriate quotes from the group discussions, and existing research. During this stage, we drew out the contextual factors (barriers and facilitators) of CVD behavioural risk factors. The reporting of this study adheres to the Consolidated Criteria for Reporting Qualitative Research (COREQ) guideline [24].

## Results

The results are presented based on the facilitators and barriers of four CVD behavioural risk factors including alcohol consumption, smoking, physical (in)activity and fruits and vegetables consumption (Supplementary Table).

### Perceptions on alcohol consumption

The narratives of the participants on facilitators of alcohol consumption (in order of dominance) focus on six main factors including psycho-social, economic, medical, socio-cultural, and sex-related factors. Participants reported that people consume alcohol in their communities when faced with different emotional responses such as worries, anxieties, sadness, hardships, broken relationships, lack of self-confidence and excitement. Their narratives showed that people drink regardless of whether they experience positive or negative emotions.

*When people are happy, they drink. When they are also facing some hardships or challenges, they drink. [Adult females, Cape Coast]*

*Some people who are shy to talk to you will go and drink alcohol so that they will be bold enough to insult or fight you. Alcohol does not make you shy. It makes people also talk a lot as if they are mad. [Elderly females, Cape Coast]*

Some groups mentioned that people consume alcohol when they experience economic challenges such as loss of job, unemployment, and poverty. At the other extreme, people consume alcohol to boost their productive capacity. Participants also mentioned that people consume alcohol for health reasons including pain suppression, illness management, improvement in sleep quality and as an appetite booster.

*There are certain diseases that consumption of alcoholic drinks could suppress. For example, when you have body pains you can take ‘adonko bitters,’ ‘ kakai bitters’, and ‘Atinka’. When you get any of these in the right amount, you could suppress your ailment… [Young males, Cape Coast]*

Further, participants reported that socio-cultural factors are important facilitators of alcohol consumption. For instance, participants, especially those recruited in Ga Mashie (Ga), mentioned that curse could be invoked on someone to indulge in alcohol consumption uncontrollably. Also, some rites performed within some of the study communities in the quest for ‘supernatural powers’ require intake of concoctions prepared with alcohol; people may indulge in excessive alcohol intake based on their level of determination to obtain these ‘supernatural powers.’ Some of the participants mentioned that people consume alcohol to boost their sexual performance. Participants noted that the built environment also favours alcohol consumption as several alcoholic beverages are available and easily accessible within the study communities, and these invariably promote excessive alcohol consumption. Particularly, participants mentioned several alcoholic beverages consumed within their communities and these can be classified under four broad categories: spirits, local drinks, beers and bitters (Table 2). There was age variation with respect to the list of alcoholic beverages consumed in the study communities; alcoholic beverages classified as bitters were mostly mentioned by young males and females in the five study communities.

**Table 2:** Categories and types of alcohol consumed in the five communities

**Categories**	**Types**
Spirits	Local gin, Shandy, 8 pm, Jack Daniels, Castle bridge, Smirnoff and Whisky, Striker gin, Choice, Capital One, Gin-kiss, and Fighter
Local drinks	Sugarcane drink, Palm wine, and Pito
Beers	Guiness, Kiss, cider, and Beers
Bitters	Abe Nsuo, Adonko, Agya Appiah, Agya Twedie, Alomo, Atemuda, Bie Gya, Herbafrik, Joy, Joy Daddy, Joy Twedie Ginger, Kakai, Kama Me, Twa Bi Mami, Twedie, Mr Osei Nana Takyi Bitters, Orijin, Kamame, Kasapreko, Fame Kor, Ginseng, Ginger, And Happy Man, Rossi Rosso, Bitters Striker

Further, the symbolic role of alcohol within the study communities was mentioned as a key factor driving alcohol consumption. First, participants mentioned that people indulge in excessive alcohol consumption during festive occasions (e.g. Christmas, Easter and during Valentine’s day), social events (e.g. funerals, traditional festivals, naming ceremonies, weddings, and customary rites such as pouring of libation, enstooling a chief, etc.), and political events (e.g. during political rallies and campaigns). Second, participants noted that alcohol performed two major roles within their communities, and these include economic and social-cultural roles. With respect to economic roles, participants mentioned that alcohol creates wealth for the sellers. Also, alcohol plays a major role during social events such as marriage rites, naming ceremonies, festivals, funerals and for invoking curse on people. According to the participants, alcohol performed a symbolic role in the settling of disputes between two groups or individuals and in seeking permission before entering some houses as a visitor.

*when there’s a dispute between two people a drink can be used to seal the settling of the misunderstanding; wrong doers are also fined with drinks; drinks are used during marriage rites. So, there are various uses of alcohol [Elderly Males, Keta]*

With respect to the effects of alcohol consumption, participants’ narratives focused on three main areas: chronic diseases; general health issues, and “socio-economic downgrade”. Participants mentioned that alcohol consumption causes chronic conditions such as kidney disease, liver disease, diabetes, hypertension, stroke, heart attack, mental disorders, and other chronic ailments. Regarding general health issues, many of the participants mentioned that alcohol intake could result in poor changes in physical appearance, weight gain, destruction of internal body organs, waist pain, diarrhoea, strength reduction, sexual weakness, anaesthesia wear-off, and general body weakness. In addition, participants said that alcohol consumption could result in socio-economic downgrade thus leading to reduction in socio-economic condition to a lower status.

### Perceptions on smoking

Three main factors emerged from the discussions across the five communities as facilitating smoking. These include (in order of dominance) personal and social acceptability, health reasons, and economic factors. Participants revealed that people engage in smoking for pleasure while others smoke because of pressure from their peers. They noted that smoking is a new trend which people follow or adopt (especially the youths), a part of living for some people, and as a motivation for students to study for long hours. Further, participants mentioned that people engage in smoking as a coping mechanism, to boost their self-confidence and as a response to emotional extremes such as sadness, depression, and excitement.

The belief that smoking has some healing properties facilitated smoking. Some of the participants believed that smoking helps in the treatment of certain medical conditions and also associated with prolonging life, improving appetite, and increasing physical strength of an individual. Furthermore, participants’ discussions revealed that economic reasons such as unemployment or poverty also trigger smoking. Participants cited cigarette, marijuana, cocaine, ‘sheesha’, and ‘pepper’ as the substances being smoked in the five communities. The narratives also showed that some community residents smoke because it is accessible and affordable.

*for smoking, nowadays it isn’t that difficult to find a young person smoking like before. But for some people, it is a medical advice to smoke. My brother for instance used to have frequent eye problems and he was advised by a certain doctor to be smoking a little cigarette. So now he smokes it so much. [Adult males, Keta]*

The circumstances under which community residents in the study communities engage in smoking can be summarised under two main categories: social events and a routine practice. Participants from central region (Akan) mentioned a social event that is carried out every year (11^th^ may), commemorated as ‘Bob Marley Day’. On this day, there is mass smoking, and people usually come out and smoke on the street. Participants mentioned that this event encourages smoking in the region. Participants, especially the young women across the five study communities, mentioned that people engage in smoking as a routine practice- an activity that people engage in on a daily basis. There were contradictory views regarding the roles of smoking. While some of the participants mentioned that smoking does not have any community function, others believed smoking performed some functions within a community. These roles can be broadly categorised into economic and psychosocial roles. According to some of the participants, smoking creates wealth for those who sell the substances but at the same time can result in socio-economic downgrade for people who smoke. Some of the participants mentioned that smoking gives peace of mind, strength or motivation to people who smoke for them to be able to work for longer periods.

Further, participants’ narratives showed that smoking had some health-related effects, and these discourage some people from smoking. These health-related effects can be categorised into three- physical health, mental health, and non-specific health related issues. Participants mentioned that smoking causes health deterioration, chronic illness, lung related comorbidities, stroke, kidney disease and tuberculosis. Additionally, participants noted that smoking can cause changes in skin colour and the physical appearance of the smoker, and can result in neglect of personal hygiene, hair loss, low sperm count and ultimately ruin someone’s life through ill health, picking up bad habits like stealing and eventually endangering community living.

*Smoking is even worse. When you become addicted to it, you lose sense of yourself. It changes your complexion and your behaviour changes. Sometimes you behave like a mad man... [R.1 Adult females, Cape Coast]*

### Perceptions on physical (in)activity

Health benefits was the only factor cited by participants as the reason why people engage in physical activity. Participants mentioned that engaging in physical activity reduces stress, minimises the effect of an illness, improves blood circulation, sleep, and digestion. They mentioned 19 different activities that were considered as physical activity, and these came up in all group discussions regardless of age and gender. These activities can be classified under two broad categories: 1) Endurance activities (walking, visiting the gym, running, press-ups, keep-fit activities, football, cycling, Squat, skipping, jumping, and swimming), and 2) Activities of Daily Living (ADL) (job related activities, household chores, dancing, clean-up exercises, singing, Games, stretching and sex).

Three factors were mentioned by participants as reasons why people do not engage in physical activity; these include 1) personal/social reasons, 2) economic reasons and 3) negative health effects. With respect to personal/social reasons, participants noted that people do not engage in physical exercise because of time constraints, personal dislike, lack of knowledge about the benefits of exercise, pride, and laziness. Personal attributes like medical conditions, age, and getting tired easily were also cited as barriers to engaging in physical activity. Participants from Ga Mashie particularly noted that community members’ perception about what loss of weight connotes prevents some women from engaging in any form of physical activity.

*…. so, this place we can’t do exercise. When you slim, they will say you’ve gone to do abortion [Young females, Ga Mashie]*

Economic hardships such as poverty, life’s challenges, and lack of food prevent people from engaging in physical activity.

*Poverty. When someone gets up in the morning, what he’s preoccupied with is not jogging or walking. He’s thinking about what to eat in the morning [Adult males, Cape Coast]*

*for some people it’s too much thinking. As a human being if you are faced with many problems or financial hardships. It will be difficult for you to wake up and tell yourself you are going to exercise. After exercising and you get hungry, what food will you eat? So, if you don’t exercise you will be a bit okay [Adult females, Kumasi*]

### Perceptions on fruit and vegetable consumption

Improvements in health status was the only factor that facilitated the intake of fruits and vegetables, as reported by the participants. Participants mentioned that intake of vegetables benefits the circulatory system by lowering blood pressure and improving the volume and circulation of blood. Furthermore, participants noted that adequate intake of fruits and vegetables enhances physical appearance, protects vision, strengthens bones, and protects teeth from decay. Participants mentioned that fruits and vegetables support weight loss and enhances digestion by preventing constipation.

Intake of fruits and vegetables was also facilitated by the variety of fruits available in the study communities. Participants listed 28 and 26 different types of vegetables and fruits, respectively which were available on the market and in their respective communities. These vegetables and fruits are available all year round, but the prices rise and fall depending on the season. The vegetables include Carrot, Cabbage, Garden eggs, Okra, Tomatoes, Ayoyo, Kontomire, Alefu, Cucumber, Green pepper, Onion, Pepper, Boabab leaves, Pear, Dandelion, Kenaf, Lettuce, Spinach, Bra, Cauliflower, Dry Okra, Bera, Dawadawa, Green peas, Spring Onion, and water leaf. The 26 fruits mentioned by participants can be classified into 1) Seasonal fruits (Neem, Ebony fruits, Shea Nuts, Mango, Watermelon, Black berries, Sugar cane, Yellow melon, Yellow berries, Soursop, Starfruit, Tangerine, red berries, Saloon Mango, Cocoa, Cocoa nut, Wild fruit, sweet apple and guava) and 2) non-seasonal fruits (Pawpaw, Apple, Pineapple, Grapes, Banana, Coconut and Orange).

Personal and economic factors were the main barriers to intake of fruits and vegetables in the study communities. Personal factors such as personal dislike, unwillingness to travel to the market for fruits, lack of knowledge about benefits of fruits and vegetables, cultural practices on consumption of certain fruits or vegetables and low satiety of fruits and vegetables limits the intake of fruits and vegetables. Apart from the group discussions in Agorve and Chanshegu communities, the relatively high cost of fruits and vegetables which contributed to its low intake dominated the economic factors in the remaining four communities. Availability of fruits and vegetables all year round, judicious use of financial resources, added preservatives, and insufficient money also came up in the participants’ narratives as barriers to intake of fruits and vegetables.

*It’s all about money. It’s when you have money that you buy. One needs to have at least 1 cedi to buy watermelon. This same amount of money can buy you two balls of kenkey. You will be satisfied if you buy the kenkey, but the fruits will not satisfy you. [Adult females, Cape Coast]*

## Discussion

This study explored perceptions of community members’ on the contextual factors driving cardiovascular disease behavioural risk (alcohol consumption, smoking, physical inactivity and fruits and vegetables consumption) in five low-resource communities in Ghana. The study particularly highlighted the facilitators of and barriers to engaging in these CVD risk behaviours and the age and gender variations in some of the perceptions of community members. Four main factors were raised by participants as the contextual factors driving CVD behavioural risk factors in these communities and these include economic, psycho-social, medical, and socio-cultural factors. The symbolic functions of some of these CVD behavioural risk factors were important determining factors that have sustained these behaviours in these communities over the years. Our study showed that participants’ knowledge on the association between CVD risk behaviours and cardiovascular disease was generally poor, and this is in line with what previous studies have shown [22, 23].

As shown by other studies, our study showed that the key factors driving excessive consumption of alcohol include psycho-social, economic, medical, easy availability of a wide range of alcoholic beverages, peer pressure, and cultural practices that support alcohol consumption in these communities [23, 25, 26]. Funeral was the main cultural practice that facilitated the consumption of alcohol in the study communities. This is not surprising, considering the elaborate ceremonies that surround funeral celebrations in Ghana, because of its social and cultural significance [27]. In Ghana, a funeral celebration is a way to honour the dead and to aid in the smooth transition from the ‘earthly’ realm to the next, and alcohol is deemed to play an important role in this [28]. Neglecting the symbolic role that alcohol play during funerals in the study communities may undermine intervention strategies to minimise the intake of harmful alcohol consumption in these communities. Similar to other studies in Ghana and sub-Saharan Africa [25, 29], participants’ knowledge on the association between alcohol consumption and cardiovascular disease was generally poor. Although many of the participants acknowledged that alcohol consumption causes other health complications, very few associated it with cardiovascular disease.

This study also showed that the key factors facilitating smoking include personal and social acceptability, health, and economic factors. Participants’ narratives suggested that smoking is gradually becoming a socially acceptable practice in cultures where such practices were once frowned upon. This finding is consistent with other studies in SSA which showed that smoking is increasing in the region and this trend could be associated with urbanisation and modernisation [26, 30]. Our findings further showed that participants’ knowledge on how smoking is associated with cardiovascular disease was poor because majority of respondents could not associate smoking to CVD but rather to mental illness, lung cancer and other lung comorbidities. This further explains why some of the community residents mentioned that smoking provides psycho-social and health benefits, necessitating the need to intensify health education in these communities.

Our findings showed that the main reason people engage in physical activity was due to its health benefits. There was a good knowledge on the health benefits of physical activity accompanied by a wide and varied list of activities considered as physical activity. This can be attributed to the fact that participants have not really understood what qualifies as physical activity which is similar to what was found in a qualitative study in Nairobi where participants mentioned weight lifting as exercise that helps reduce CVD instead of aerobic exercises [25]. A plausible explanation for this may be because these are mostly low-resource communities that engage in daily physical activities such as agriculture and hawking which they find physically exhausting [23].

Participants mentioned that the barriers to engaging in physical activity in their communities include personal/social reasons, economic reasons and negative health effects, which is in line with what other studies have shown [23, 31, 32]. However, this finding is in contrast to another study in Nairobi where the main barrier to physical activity was crime and insecurity in the neighbourhood [29]. Cultural ideas that associate increase in body weight with wealth or a sign of being well catered for by spouse or significant other was brought out among young women groups and this in line with other studies [25, 26, 30, 32]. However, this is contrast with an earlier study done in sub-Saharan Africa that revealed that women are interested and willing to lose weight to reduce the risk of CVD as opposed to community or husband’s preference [33]. This contrast could be due to personal attributes or background characteristics of the respondents used in the different studies.

The key facilitator of consumption of fruits and vegetables is the health benefits [23] and the variety of fruits and vegetables available to respondents. However, in line with other studies in Ghana and SSA, the cost of fruits and vegetables were mentioned as a barrier to the consumption of fruits and vegetables. These studies showed that cost and availability were key factors that affected healthy food choices [20, 22, 25]. Congruent with [25] participants avoided foods that have very low satiety which was also a barrier to the consumption of fruits and vegetables in order to reduce food expenditure. The plausible reason is that the community enhances intake of local staple foods which is available and affordable to its members as compared to healthier options like fruits and vegetables [25]. There is a need for the government to intensify programmes that encourage backyard gardening in houses which will encourage intake of fruits and vegetables. Furthermore, there is the need to develop culturally sensitive ways that encourage and enhance cultural values in educating the public about cardiovascular risk behaviours.

### Limitation

This study used a convenience sample; hence, the findings are not generaliseable to all Ghanaian communities or ethnic groups. However, this study provided insight on perceptions of community members on CVDs behavioural risk factors in Ghana and highlighted important contextual factors to consider in implementing CVD interventions in the country.

## Conclusion

CVDs risk factors are increasing in Ghana and other countries in SSA, and current projections suggest a further increase in the next decade. This study has importantly shed light on the contextual factors driving CVD behavioural risk factors in Ghana and showed the importance of addressing these contextual factors for successful implementation of CVD interventions to take place in the country. Future policies and interventions should be developed based on the above insights especially interventions seeking to address CVD risk factors at the primary health care level. We anticipate that these findings should urge local policy makers and health managers to incorporate the roles of these contextual factors and how these roles are shaped by age, gender and ethnic disparities in new programs targeting cardiovascular health. Closing the ‘evidence to practice’ gap as far as CVD interventions are concerned may be impossible without this.

## Supplementary Information

**Additional file 1: Supplementary Table 1.** Contextual factors driving CVD behavioural risk factors.

## Data Availability

The datasets used and /or analysed for this study are not available on a public repository as they contain identifiable and sensitive information making it impossible to protect participants’ confidentiality. The datasets are available from the corresponding author on reasonable request.

## Abbreviations

CVD: Cardiovascular disease
NCD: Non-communicable diseases
FGDs: Focus Group Discussions
HIC: High Income countries
LMIC: Low- and middle- income countries
SSA: Sub-Saharan Africa
ADL: Activities of daily living
