# Correction: Perceptions of community members on contextual factors driving cardiovascular disease behavioural risk in Ghana: a qualitative study

**DOI:** 10.1186/s12889-022-13704-w

**Published:** 2022-08-10

**Authors:** Naa Adjeley Mensah, Olutobi Adekunle Sanuade, Leonard Baatiema

**Affiliations:** 1 Regional Institute for Population Studies, University of Ghana, Accra, Ghana; 2 Department of Medical Social Sciences, Northwestern University Feinberg School of Medicine, Chicago, USA; 3 Department of Health Policy, Planning and Management, School of Public Health, University of Ghana, Legon, Accra, Ghana

**Correction: BMC Public Health 22, 1240 (2022)**

**https://doi.org/10.1186/s12889-022-13646-3**

In the original publication [1] there was an incorrect funding acknowledgement. In this correction article the correct and incorrect funding acknowledgement are published. The original article has been updated.

**Incorrect funding**

- This study was supported by the Wellcome Trust [**106534**]. The funding body had no role in the study design and collection, analysis, and interpretation of data and in writing the manuscript.

**Correct funding**

- This work was supported by the Wellcome Trust [**106534/Z14/Z**]. The funding body had no role in the study design and collection, analysis, and interpretation of data and in writing the manuscript.
