# Supplementary material for: Perceptions of community members on contextual factors driving cardiovascular disease behavioural risk in Ghana: a qualitative study
Source: BMC Public Health. 2022 Jun 22;22:1240. doi: 10.1186/s12889-022-13646-3 (PMC9219153; doi:10.1186/s12889-022-13646-3)
Supplement: Supplementary file 1 — Additional file 1: Supplementary Table 1. Contextual factors driving CVD behavioural risk factors. [file 12889_2022_13646_MOESM1_ESM.docx]

**Supplementary Table l: Contextual factors driving CVD behavioural risk factors**

| **Determining factors** | **Alcohol** | **Smoking** | **Physical inactivity** | **Fruits and vegetables consumptions** |
| --- | --- | --- | --- | --- |
| ***Reasons for engaging in lifestyle behaviours*** |  |  |  |  |
| Psycho-social factors (e.g. worries, sadness, excitement, etc) | * | * |  |  |
| Economic (e.g. loss of jobs, unemployment, etc) | * | * |  |  |
| Health reasons (e.g. pain suppression, health and illness management, etc) | * | * | * |  |
| Socio-cultural (e.g. curse, to obtain supernatural powers, etc) | * | * |  |  |
| Sexual (e.g. to boost sexual performance) | * |  |  |  |
| ***Circumstances under which people indulge in these lifestyles*** |  |  |  |  |
| Social events (e.g. funerals, naming ceremonies, etc) | * | * |  |  |
| Festive seasons (e.g. Christmas, Easter,etc) | * |  |  |  |
| Political events (e.g. political rallies and campaigns) | * |  |  |  |
| Routine (Daily, Weekends etc) |  | * |  |  |
| ***Effects of poor lifestyle behaviours*** |  |  |  |  |
| Result in chronic diseases (e.g. stroke, diabetes, hypertension, etc) | * | * |  | * |
| Causes general health issues (e.g. weight gain, sexual weakeness, waist pain, etc) | * | * |  | * |
| Leads to socio-economic downgrade (e.g. poverty) | * |  |  |  |
| Leads to changes in mental state |  | * |  |  |
| **Roles of lifestyle behaviours within the community** |  |  |  |  |
| Economic role (e.g. wealth creation for sellers) | * | * |  |  |
| Socio-cultural (e.g. dispute settlement, symbolic during social events) | * |  |  |  |
| Psychosocial (eg peace of mind, stealing, improving appetite, etc) |  | * |  |  |
